# Visceral artery pseudoaneurysms: two case reports and a review of the literature

**DOI:** 10.1186/s13256-017-1291-6

**Published:** 2017-05-04

**Authors:** Amna Abdelgabar, Olivier d’Archambeau, Joachim Maes, Filip Van den Brande, Peter Cools, Roger R. Rutsaert

**Affiliations:** 1 Department of Vascular and Thoracic Surgery, Sint Vincentius Hospital, Antwerp, Belgium; 2 Department of Radiology, Sint Augustinus Hospital, Antwerp, Belgium; 3 Department of General Surgery, Sint Vincentius Hospital, Antwerp, Belgium

**Keywords:** Case report, Endovascular, Interventional radiology, Visceral pseudoaneurysm, Superior mesenteric artery, Gastroduodenal artery

## Abstract

**Background:**

Visceral artery pseudoaneurysms are relatively rare but have a high mortality rate in case of rupture. Their detection in the last decades is rising due to an increased use of computed tomography and angiography. However, due to the nonspecific nature of the clinical symptoms and signs, diagnosis is often delayed or missed. We describe two cases of patients presenting with nonspecific abdominal complaints and anemia leading to a diagnosis of visceral pseudoaneurysm. Both cases are successfully treated with a different endovascular intervention.

**Case presentation:**

The first case is a 67-year-old Caucasian man presenting with diffuse abdominal pain, vomiting, diarrhea, and weight loss. Digital angiography showed a complex pseudoaneurysm of the superior mesenteric artery. The patient was treated with stent placement and selective embolization of the afferent branches.

The second patient is a 78-year-old Caucasian man with a history of chronic pancreatitis admitted with epigastric pain, rectal bleeding and melena. Angiography showed a pseudoaneurysm of the gastroduodenal artery. The patient was successfully treated with coil embolization.

**Conclusions:**

We report two cases of visceral pseudoaneurysms and review the literature concerning etiology, presentation, diagnosis, and treatment.

Visceral artery pseudoaneurysms should be considered in the differential diagnosis of a patient with nonspecific abdominal symptoms. Diagnosis is often made with computed tomography or computed tomography angiography but digital angiography remains the gold standard. Treatment options include surgical, endovascular or percutaneous interventions. The choice of treatment is case specific.

## Background

Visceral artery aneurysms (VAAs) and pseudoaneurysms (VAPAs) are relatively rare with a reported incidence of 0.01 to 0.2% in routine autopsies [1, 2]. The splenic artery is the most common site of aneurysmal disease (60%), followed by the hepatic arteries (20%), superior mesenteric artery (5.5%), coeliac trunk (4%), gastric and gastroepiploic arteries (4%), intestinal arteries (3%), pancreaticoduodenal arteries (2%), gastroduodenal artery (1.5%), and the inferior mesenteric artery (1%) [2].

We present two cases of visceral pseudoaneurysms. The first case is a patient with a complex pseudoaneurysm of the superior mesenteric artery (SMA) treated with stent placement and selective embolization of the afferent branches. The other patient presented with a pseudoaneurysm of the gastroduodenal artery (GDA) treated with coil embolization. A review of the literature concerning etiology, presentation, diagnosis and treatment is discussed.

## Case presentation

### Case 1

**Fig. 1:**
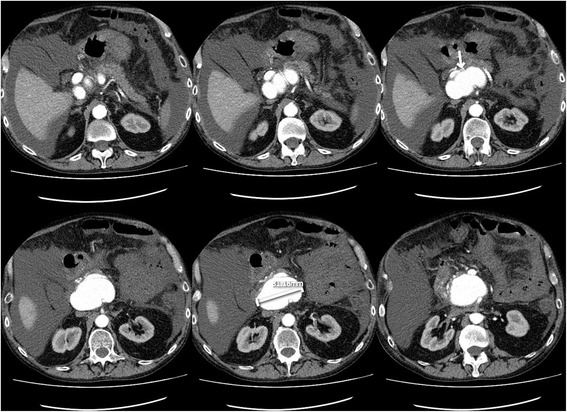
Preoperative computed tomography angiography showing a large pseudoaneurysm between the superior mesenteric artery and coeliac trunk with a diameter of 81 mm. The *arrow* points to a portal fistulization

**Fig. 2:**
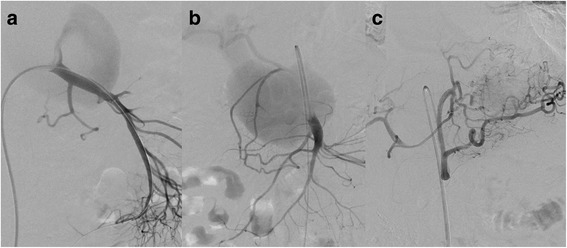
Preoperative angiography showing a pseudoaneurysm of the proximal superior mesenteric artery (**a**, **b**) with fistulization to the portal vein (**b**). The pseudoaneurysm is filled partially via a large defect in the top of the superior mesenteric artery (**a**). There is no connection between the common hepatic artery and the coeliac trunk (**c**)

A 67-year-old Caucasian man was admitted with diffuse abdominal pain, vomiting, diarrhea, and 4 kg weight loss within a couple of weeks. He had a history of alcohol abuse but further medical history was negative. There was no significant abdominal tenderness, no abdominal palpable mass or flank-knocking tenderness. Laboratory studies showed mild anemia with a hemoglobin level of 10.0 g/dL. An abdominal ultrasound (US) scan showed a partially inverted flow in the portal vein with a small cirrhotic liver, ascites, and a small spleen. Abdominal computed tomography (CT) revealed a large pseudoaneurysm between the SMA and coeliac trunk with a diameter of 77 × 53 × 59 mm. Just below this pseudoaneurysm lay the pancreas with calcifications due to chronic pancreatitis. CT angiography revealed an arterioportal fistula between the pseudoaneurysm and portal vein. Due to this pseudoaneurysm, there was compression of the superior mesenteric vein. Ascites around the liver and spleen were seen, probably due to portal hypertension. There was an increase of the pseudoaneurysm diameter compared to the abdominal CT scan made 5 days earlier, with a maximal diameter of 81 mm (Fig. 1). Digital angiography revealed a pseudoaneurysm arising from the proximal superior mesenteric artery with fistulization to the portal vein. The pseudoaneurysm was filled via a large defect in the top of the superior mesenteric artery (Fig. 2a, b).

**Fig. 3:**
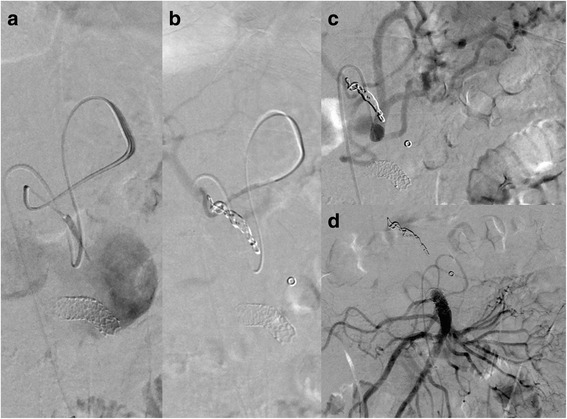
Postoperative angiography showing a covered stent (Bentley Begraft® diameter 8 mm, length 37 mm) covering the defect in the superior mesenteric artery and refilling of the pseudoaneurysm via the common hepatic artery (**a**). Embolization of the common hepatic artery with multiple Hilal microcoils (3, 4, and 5 mm wide and 3 and 6 cm long) after selective catheterization of the common hepatic artery via the left and then the right gastric artery (**a**, **b**). Complete exclusion of the pseudoaneurysm sac after coiling of the common hepatic artery and covered stenting of the superior mesenteric artery (**c**, **d**)

From a technical point of view, covered stenting of the SMA was preferred over embolization of the pseudoaneurysm. A 6 French LIMA sheath was placed in the superior mesenteric artery using a femoral approach. Next, a balloon-expandable covered stent (Bentley Begraft® diameter 8 mm, length 37 mm; Bentley, Hechingen, Germany) was deployed in the SMA. Control angiography showed refilling of the pseudoaneurysm via the common hepatic artery (CHA) through the right gastric and gastroduodenal arteries (Fig. 3a). Since there was no filling of the CHA from the coeliac trunk, it is probable that the CHA originated from the SMA as a normal variant and was separated from its origin, possibly due to the pancreatitis (Fig. 2c). After catheterization of the CHA via the left and then the right gastric artery, selective embolization was performed with a 2.7 Fr Progreat® microcatheter (Terumo Medical Corp., Somerset, NY, USA) using multiple Hilal microcoils (3, 4, and 5 mm wide and 3 and 6 cm long; Cook Medical, Bloomington, IN, USA), covering the origin of the GDA (Fig. 3b-d). The residual perfusion of the liver is only supplied by the right gastric artery from the left gastric artery.

Postoperatively, the patient was given clopidogrel therapy for 6 weeks and lifelong acetylsalicylic acid.

**Fig. 4:**
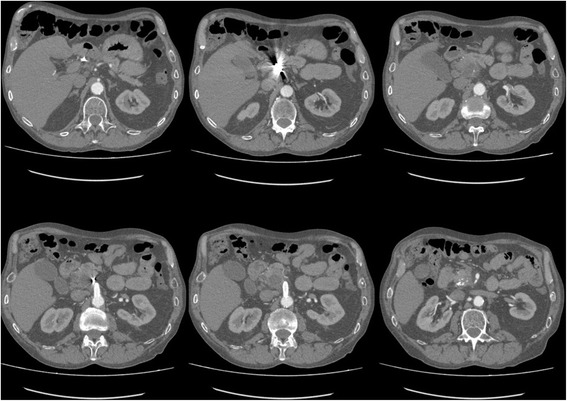
Control computed tomography angiography after 6 months shows complete thrombosis and resorption of the pseudoaneurysm. Covered stent in the superior mesenteric artery and coils in the common hepatic artery

He was discharged on the eighth postoperative day. Follow-up after 6 months revealed no recurrent abdominal symptoms. The pseudoaneurysm was no longer detectable on control CT angiography (Fig. 4).

### Case 2

**Fig. 5:**
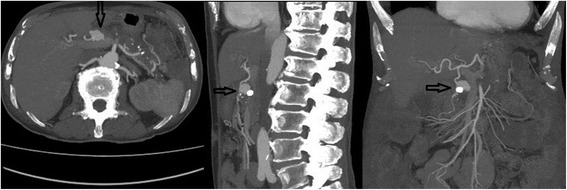
Preoperative computed tomography angiography showing a (pseudo-)aneurysm of the gastroduodenal artery of 24 mm (*arrows*). There is a clear calcification of 11 mm, compatible with calcified thrombosis. Transverse, sagittal and coronal views from *left to right*

**Fig. 6:**
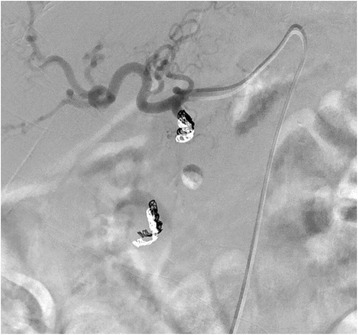
Postoperative angiography after selective catheterization of the proper hepatic artery: complete exclusion of the pseudoaneurysm sac after coiling of the gastroduodenal artery

A 78-year-old Caucasian man with a history of mucosa-associated lymphoid tissue (MALT) lymphoma and chronic pancreatitis presented with epigastric pain, rectal bleeding, and melena. Both 2 months and 2 weeks before he had also been admitted with rectal bleeding and need for transfusion. His vital signs showed tachycardia of 115 beats/minute with normal blood pressure. A physical examination was unremarkable with no abdominal tenderness. Laboratory studies showed severe anemia with a hemoglobin of 4.0 g/dL. An urgent esophagogastroduodenoscopy was performed but did not reveal the source of the bleeding. CT angiography showed a (pseudo-)aneurysm of the gastroduodenal artery of 24 mm with bleeding in the small intestine (Fig. 5). Digital angiography was performed and showed a high-grade ostial stenosis of the coeliac trunk with a rather broad superior mesenteric artery. Dissection of the gastroduodenal artery with a pseudoaneurysm at this level is seen after selective catheterization with a 2.7 Progreat® microcatheter (Terumo Medical Corp). This pseudoaneurysm was crossed and distally (to “close the back door”) a plurality of microcoils was placed. Then, the tip was placed just proximal to the neck of the pseudoaneurysm, and multiple coils were placed here (4 mm wide and 3 mm long). This “sandwich” technique prevents both downstream and upstream filling. Post-procedure angiography from the proper hepatic artery showed a good closure of the pseudoaneurysm (Fig. 6).

Our patient had no further gastrointestinal hemorrhage. He had an uneventful postoperative course and was discharged on the tenth postoperative day. Control CT angiography after 6 months showed complete thrombosis and resorption of the pseudoaneurysm.

## Discussion

### Etiology

Pseudoaneurysms are an uncommon complication of chronic pancreatitis. Sixty-eight percent of visceral pseudoaneurysms are secondary to pancreatitis and pseudocyst formation and 10 to 17% of all patients with chronic pancreatitis develop pseudoaneurysms [3, 4]. Visceral pseudoaneurysm formation is believed to be the result of leakage of proteolytic enzymes in the setting of pancreatitis with destruction of the vessel wall. They may also result from erosion of nearby pseudocysts into adjacent vessels [3, 4]. Blunt or penetrating abdominal trauma and iatrogenic trauma, for example, after hepatobiliary or vascular surgery or after pancreatic head biopsy, may also cause pseudoaneurysms [4, 5]. The proportion of pseudoaneurysms versus true aneurysms differs by arterial location [6]. In case of gastroduodenal and superior mesenteric arteries Fankhauser *et al*. reported more VAPAs than VAAs (89% versus 11% and 67% versus 33% respectively) [7].

In both presented cases, it is likely that pseudoaneurysm formation was related to the chronic pancreatitis. In the first case the pseudoaneurysm was filled partially via a large defect in the top of the superior mesenteric artery and partially via the CHA through the pancreaticoduodenal arcade, the gastroduodenal artery, and gastric artery. It was complicated with fistulization to the portal vein. In the second case, there was a dissection of the GDA with formation of a pseudoaneurysm. In addition to the pancreatitis, the high-grade ostial stenosis of the coeliac trunk probably played an underlying role in the etiology of this pseudoaneurysm.

### Presentation

Both patients presented with abdominal pain and anemia. The second patient also experienced rectal bleeding and melena. Visceral artery pseudoaneurysms are usually asymptomatic and identified as incidental, unexpected findings on imaging of the abdomen, particularly on CT or CT angiography [8]. But unlike other visceral artery (pseudo-) aneurysms (VA(P)As), 70 to 90% of SMA aneurysms and almost all SMA pseudoaneurysms are symptomatic [9].

Abdominal pain is the most common symptom of unruptured VA(P)As [10–12]. In case of rupture, a gastrointestinal hemorrhage is the most common clinical presentation [10–12]. Depending on the location it can cause hematemesis, melena, hematochezia, hemobilia, retroperitoneal hemorrhage, and hemorrhagic shock [3, 13]. The pseudoaneurysm can also rupture in the duct of Wirsung, causing a so-called hemosuccus pancreaticus [3, 4]. Pathogenesis of the bleeding is most probably related to intermittent erosion and breakdown of the aneurysm wall through the duodenal wall, common bile duct, pseudocyst or duct of Wirsung, secondary to pressure necrosis caused by the expanding pseudoaneurysm [13]. A pulsatile mass or bruit is the second most common symptom of a VA(P)A of the superior and inferior mesenteric artery in contrast to other visceral arteries [6, 11]. Other clinical symptoms of VAPAs are intraperitoneal or retroperitoneal bleeding, jaundice, compressive symptoms (nausea, vomiting) or gastric outlet obstruction [5, 6, 10–12].

### Diagnosis

Critical clues that aid in the diagnosis of visceral pseudoaneurysms include anemia of unexplained cause, recurrent and intermittent hematemesis or hematochezia, and rapid enlargement of an otherwise stable pseudocyst [4].

Ultrasonography can reveal a hypoechoic cystic lesion in close relation to an artery. Color Doppler US can display the typical inflow and outflow of blood as a swirling motion in the sac through the neck. This is known as the “yin-yang sign” [14]. CT angiography provides additional information about the pseudoaneurysm as well as the disease process [15]. Digital angiography remains the gold standard for diagnosis of pseudoaneurysms due to its capability of real-time assessment of the site of extravasation. The anatomy of the collateral vessels and the extent of the involved vessels can be evaluated. This is important when planning an endovascular procedure [4, 16]. Digital angiography has the highest sensitivity (100%), followed by CT (67%) and US (50%) [10]. In both our cases the definitive diagnosis was made with digital angiography. In the first case, CT angiography showed a pseudoaneurysm between the SMA and coeliac trunk. Digital angiography showed filling of the pseudoaneurysm via a large defect in the top of the superior mesenteric artery and via the CHA. When reviewing the facts that the pseudoaneurysm was filled through a defect proximal in the SMA and retrograde through the CHA and there was no filling through the coeliac trunk, we could suggest a normal variant with a replaced CHA arising from the SMA. In our second case, CT could not tell the difference between a true or false aneurysm. Definitive diagnosis of a dissection of the gastroduodenal artery with formation of a pseudoaneurysm was made with digital angiography.

### Treatment

According to the guidelines, treatment of a true visceral aneurysm is indicated when its diameter is >2 cm or three times greater than the respective normal artery [5, 17–19]. Treatment is also indicated in case of rapid expansion of >0.5 cm/year, symptoms, woman who are pregnant or of childbearing age, and patients undergoing an orthotopic liver transplantation [20–22]. On the contrary, a pseudoaneurysm must be treated immediately because the rate of rupture is much higher in pseudoaneurysms than in true aneurysms (76.3% versus 3.1%) [5]. Depending upon the diameter and the location, pseudoaneurysm rupture is associated with a mortality rate ranging from 25 to 70% [20].

Treatment options include surgical (arterial bypass, exclusion of the aneurysmal sac, vessel ligature), endovascular (embolization, stent placement) or percutaneous (thrombin injection) interventions. For vessels that supply an end organ without multiple sources of blood flow, patency of the feeding vessel should be preserved (either through stent placement or surgical revascularization). However, collaterals between the visceral arteries almost always exist, therefore most VAPAs can be treated by ligation or embolization.

If the different factors permit it, the less invasive endovascular interventions should be exhausted before proceeding with surgery. In case of failure, vascular surgery will still be a feasible alternative. Since endovascular treatment is less invasive and can be performed under local anesthesia, it offers a good therapeutic strategy for patients who are inoperable due to severe comorbidities.

The transcatheter selective embolization of pseudoaneurysms has become the most commonly used approach. Different materials can be used like coils, gelatin foam, polyvinyl alcohol (PVA) particles, trisacryl gelatin microspheres (TAGM), amplatzer vascular plugs (AVP), cyanoacrylate glue, ethylene vinyl alcohol copolymer (EVOH-Onyx®) or calcium alginate gel (ALGEL) [23]. One can choose between proximal embolization or, whenever possible, the “sandwich” technique to occlude the artery proximally and distally to the pseudoaneurysm to prevent anterograde and retrograde filling [3]. This technique was used in our second case.

If patency of the feeding vessel needs to be maintained, covered stents can be a good alternative treatment without losing the benefits of an endovascular intervention. However certain factors preclude their use. The length of the vessel on both sides of the pseudoaneurysm must be sufficiently long in order to ensure an adequate seal [7, 24]. A length of at least 10 mm proximal and distal of the stent is recommended [24]. Severe tortuosity or sharp angulation can make stent placement unfeasible. Hemp *et al*. recommends treating tortuous arteries with a self-expanding covered stent while straight arteries can be treated with balloon-expandable stents [24]. Using bare stents and coiling through the mesh of the stent with a microcatheter and microcoils is another alternative to preserve patency. The risk of stent thrombosis or restenosis resulting in potential end-organ ischemia must be taken into consideration. Given the complexity, location on the SMA, and size of the pseudoaneurysm, we chose to place a balloon-expandable covered stent in our first case.

No consensus has been reached regarding the duration of antiplatelet therapy after stenting. In our first case, we decided on clopidrogrel for 6 weeks and acetylsalicylic acid lifelong.

Fankhauser *et al*. reported endovascular treatment of 185 aneurysms (64% VAPAs) with a success rate of 98%. Reintervention was required in 3% within 30 days. The 30-day aneurysm-related mortality was 3.4% and the periprocedural mortality rate was 6.2% [7]. Sethi *et al*. reported a success rate of 77% after coil embolization in 14 patients with a visceral pseudoaneurysm. Persistent perfusion in three patients (20%) was effectively managed by a secondary coil embolization [25]. Both Won *et al*. and Balderi *et al*. reported a 100% success rate in 13 patients with a VAPA. Aneurysm-related mortality (*n* = 0 and *n* = 1) and morbidity (*n* = 2 and *n* = 1) was low in both studies [26, 27].

Reported complications of endovascular therapy include access-related complications such as femoral artery pseudoaneurysms, thrombosis or embolism; access-site hematoma, pain, cellulitis or infection, and technical failure to catheterize the artery. In patients with poor renal function, the possibility of contrast agent-induced nephropathy must be considered. Other potential complications are distal thromboembolism, nontarget embolism, visceral ischemia, coil or stent migration, stent occlusion, post-embolization syndrome or intraprocedural pseudoaneurysm dissection or rupture. Possible late-term complications are reperfusion or recurrent bleeding of a pseudoaneurysm [3, 4, 7, 10, 28].

Thrombin injection guided either by US or CT is another minimally invasive technique. It was first described in 2000 by Kang for the use of post-catheterization femoral pseudoaneurysms, but can also be used for visceral pseudoaneurysms [29]. A pseudoaneurysm with a small neck and relatively slower flow is best suited for this procedure due to a lower propensity for distal embolization as well as lower probability of recanalization in the early post-injection period [16, 30, 31]. Thrombin injection can also be performed endoluminally by placing a microcatheter into the aneurysm sac. A disadvantage of this technique is that thrombin is not radiopaque and distal embolization may not be recognized during the procedure [24, 31]. Other possible complications are allergic reactions and infections [31].

Open surgery is recommended in case of a hemodynamically unstable patient, failed endovascular repair or unsuited anatomy. Sachdev *et al*. and Dohan *et al*. however, suggested that hemodynamically unstable patients can be treated successfully with endovascular interventions [32, 33]. Surgical treatment includes arterial bypass, exclusion of the aneurysmal sac or vessel ligature. In some cases, end-organ resection (that is, splenectomy, bowel resection) is needed [34].

Finally, visceral pseudoaneurysms can also be treated laparoscopically. This technique has been described in true aneurysms of the splenic artery by laparoscopic artery ligation or aneurysmectomy [35, 36].

Thus, treatment options are very varied. They can be used as monotherapy or in combination. Cumbie *et al*. treated a superior mesenteric artery pseudoaneurysm using common hepatic artery to SMA bypass, exclusion of the pseudoaneurysm with ligation of the SMA proximal to the bypass, plug occlusion of the proximal SMA, and coil embolization of the pseudoaneurysm [6]. In our first case, the SMA pseudoaneurysm was treated with stent placement and selective embolization of the afferent branches.

## Conclusions

It is difficult to determine the best treatment option for a visceral pseudoaneurysm. The choice of the procedure depends on the involved artery, the localization of the pseudoaneurysm, general condition of the patient, the urgency of intervention, the risk of organ ischemia after the intervention, and the experience of the physician [34, 37]. Review of the literature shows that endovascular treatment offers a good and less invasive alternative to conventional surgical intervention.
